# Our Hospitals: Their Future Organisation and Work

**Published:** 1913-08-02

**Authors:** 


### The Hospital

The Workers' Newspaper of Administrative Medicine and Institutional Life, Administration, National Insurance and Health.

No. 1414, Vol. LIV. SATURDAY, AUGUST 2, 1913. PRICE ONE PENNY

### OUR HOSPITALS: THEIR FUTURE ORGANISATION AND WORK.

The Medico-Sociological Section at the meeting of the British Medical Association at Brighton was probably the most successful of all. Its practical utility, both in regard to the consideration of "Crime and Punishment," and especially of "Hospitals in Relation to the State, the Public, and the Medical Profession," was marred, however, by the method followed at these meetings, which results in many men of experience regarding participation in them by paper or speech as a loss of time. This is due to the failure of the executive to place the papers in the hands of those who are invited to take part in the discussions a few days before the meetings are held. The result is that those who attend the meetings have first to listen, as in the Hospitals Section this year, to several papers, not one of which has been made known beforehand; this is unjust to writers and handicaps intending speakers very seriously indeed. A notification is made to the press to the effect that no paper is allowed to be published in full, as it is the property of the *British Medical Journal*. If this is why papers are withheld from the speakers invited to discuss the various questions, one of two things must result. Either the view held by the leading authorities we have already referred to will become general, and the discussions at the British Medical Association meetings must continue to fail to be of the practical value they ought to be, or the rule in question must be modified to the extent of supplying a copy of each paper to the speakers at least one day before the day of meeting. Possibly the rule quoted accounts in large measure for the relatively small amount of space which the leading London dailies devote at present to the proceedings of the meetings of the British Medical Association.

Turning now to "Hospitals in Relation to the State, the Public, and the Medical Profession," as dealt with at Brighton, we have a striking instance of the need for reform in the procedure adopted by the British Medical Association. It had been arranged and notified that the proceedings would be adjourned for luncheon, and that the discussion would be opened in the afternoon by a speech dealing with the questions raised as they affected the British hospital system. In the result several of the audience went away under this impression, and some surprise was caused by the announcement about half-past twelve that the discussion would not be continued in the afternoon. We, of course, have no knowledge why this step was taken, but we have had an intimation of the disappointment which it caused, and it no doubt contributed to destroy some of the practical usefulness of the proceedings in regard to hospitals at Brighton.

We give a full abstract of the papers, with a report of the speeches, in another column. Professor Moore's was undoubtedly the most interesting and suggestive of the papers; on the whole, we are in agreement with much he urged, and where we differ from him we believe the ground of objection is due to a fuller knowledge of the facts which govern the present hospital position, and which must continue to govern the future organisation and work of the hospitals as part of a national system connected with a perfected department of health. For instance, we have never encountered the statement which Professor Moore condemns that ethical training effected by charity and voluntary effort is a ground for opposing a national scheme to secure co-operation and intercommunication between voluntary, Government, and municipal institutions for the relief of the sick. Dr. Moore seems under the impression that the Bill of the City of Liverpool now before Parliament to authorise grants to the funds of two voluntary hospitals embodies a novel principle for Liverpool. At the Oxford Conference Mr. Deacon, the chairman of the principal voluntary hospital affected, stated that Liverpool has made grants of this kind to voluntary hospitals for fifty years, and the city has power to appoint a representative on the hospital committee. The annual subscribers to Liverpool Infirmary like to get as much money as possible from wherever they can obtain it. When, however, Professor Moore advocates that the management of all hospitals should be handed over to local public control in each district, he seems to us to advocate something which experience has proved must, if adopted, destroy the voluntary hospital system and change the present system of managing our hospitals for something which has proved already in this country to lead to grave abuses and defects, and the relegation of the needs and claims of individual patients, now so safeguarded and protected, to oblivion.

There is no need whatever for introducing this bugbear of State control of voluntary hospitals, which is recognised by politicians of both parties and by philanthropists and hospital authorities in the first rank as being something which the people of Great Britain will never suffer. In a word, the people of England would not endure a system which changed our present hospitals and made each of them a centre where science rules, and these institutions will be made "the pulsating life-centre of a little corps charged with the maintenance of the highest degree of life in a given district and co-related to similar corps and units all over the country." Everything material and useful which Professor Moore advocates can be secured by utilising the whole of our present hospitals of every type—voluntary, municipal, and State—and bringing them into a combination to aid one another in the evolution of the best possible hospital and health system the world has ever seen. Such a combination would preserve the best features of our present voluntary hospitals, where science was properly subordinated to the interests, comfort, and well-being of the patients, and these principles would constitute the life-blood of a perfected organisation, which would make the work of the hospitals part of a national scheme for the combat of disease.

